# No Association between Ischemic Stroke and Portal Vein Thrombosis in Liver Cirrhosis

**DOI:** 10.1155/2020/8172673

**Published:** 2020-07-01

**Authors:** Kexin Zheng, Xiaozhong Guo, Fangfang Yi, Le Wang, Andrea Mancuso, Xingshun Qi

**Affiliations:** ^1^Liver Cirrhosis Study Group, Department of Gastroenterology, General Hospital of Northern Theater Command (Formerly General Hospital of Shenyang Military Area), Shenyang, Liaoning Province, China; ^2^Postgraduate College, Jinzhou Medical University, Jinzhou, Liaoning Province, China; ^3^Postgraduate College, Dalian Medical University, Dalian, Liaoning Province, China; ^4^Medicina Interna 1, Azienda di Rilievo Nazionale ad Alta Specializzazione Civico-Di Cristina-Benfratelli, Piazzale Leotta 4, 90100 Palermo, Italy

## Abstract

**Background and Aims:**

There seems to be a higher risk of ischemic stroke and portal vein thrombosis in liver cirrhosis. Both of them may be associated with hypercoagulability. We aim to explore the association between ischemic stroke and portal vein thrombosis in liver cirrhosis. *Study Design and Methods*. We selected patients from our prospectively established database of liver cirrhosis from December 2014 to July 2019. The difference between patients with and without stroke was compared. A 1 : 1 propensity score matching (PSM) analysis was performed to adjust the effect of age, sex, Child-Pugh score, and MELD score on our statistical results.

**Results:**

There were 349 cirrhotic patients in the cross-sectional study. The prevalence of stroke, ischemic stroke, hemorrhagic stroke, and portal vein thrombosis was 8.88% (31/349), 8.31% (29/349), 1.15% (4/349), and 28.65% (100/349) in liver cirrhosis, respectively. Patients with ischemic stroke were significantly older and had significantly higher proportions of alcohol abuse, smoking, and arterial hypertension and higher levels of white blood cell and low-density lipoprotein. However, statistical analyses with and without PSM did not find any significant association between ischemic stroke and portal vein thrombosis in patients with liver cirrhosis.

**Conclusion:**

Ischemic stroke might not be associated with portal vein thrombosis in liver cirrhosis.

## 1. Introduction

Stroke, an acute cerebrovascular disease, is the leading cause of death and disability worldwide, especially in Asia [1]. It has been traditionally considered that stroke, especially atherosclerotic ischemic stroke, is closely associated with hypercoagulability, such as increased homocysteine and lipoprotein(a) and antiphospholipid antibodies [2]. Liver cirrhosis, which often has an increased level of factor VIII (FVIII) and decreased level of protein C (PC) and mean lifetime of platelet, has been also considered as a potential risk factor of stroke [3, 4]. Besides, it is associated with the development of venous thromboembolism, including portal vein thrombosis [5].

Development of stroke and portal vein thrombosis in liver cirrhosis seems to share several common pathophysiological mechanisms or risk factors. First, both of them relate to the hypercoagulability of cirrhosis [3], which is characterized as reduced PC in combination with increased FVIII [6]. Second, both stroke and portal vein thrombosis relate to portal hypertension. Reduced portal vein flow velocity and portosystemic collateral shunt are common characteristics of portal hypertension, which aggravate the development of portal vein thrombosis [7]. Ascites and esophageal variceal bleeding are major clinical manifestations of portal hypertension, which result in the decrease of effective circulating blood volume and hypovolemia of organs, thereby leading to ischemic stroke [8]. Third, diabetic patients have approximately 2-4 times higher risk of ischemic stroke than those with normal glucose levels [9]. Meanwhile, diabetes is also an independent risk factor of portal vein thrombosis [10]. Fourth, antiphospholipid syndrome may increase the risk of ischemic stroke as well as portal vein thrombosis. Irregular thickening of the valve leaflets secondary to antiphospholipid syndrome and lupus anticoagulants are significant risk factor of stroke [11]. Meanwhile, anticardiolipin antibodies levels were higher in liver cirrhosis with portal vein thrombosis than that without portal vein thrombosis [12, 13]. Fifth, Helicobacter pylori not only acts as a promoter of antiphospholipid syndrome through chronic inflammation [14] but also stimulates the production of plasminogen activator inhibitor-2, which has an effect on increasing the risk of ischemic stroke and portal vein thrombosis [15]. Sixth, methylenetetrahydrofolate reductase (MTHFR) mutation is a common risk factor of stroke and portal vein thrombosis, which relates to endothelial damage [16]. MTHFR activity is reduced in the patients with MTHFR mutations, thereby leading to the deficiency of folate and hyperhomocysteinemia. MTHFR is responsible for catalyzing the reduction of 5,10-methylenetetrahydrofolate to 5-methyltetrahydrofolate in the folate cycle, which further produces the active form of folate for the remethylation of homocysteine to methionine [17]. There is a significant association between hyperhomocysteinemia and stroke [18]. An elevated homocysteine concentration significantly increases the risk of stroke [19]. Folic acid supplementation is effective in stroke prevention [20]. And MTHFR A1298C mutation is associated with increased risk of ischemic stroke [21]. On the other hand, MTHFR C677T mutation may increase the risk of portal vein thrombosis in cirrhotic patients [22]. The prevalence of hyperhomocysteinemia is significantly higher in cirrhotic patients with portal vein thrombosis than those without portal vein thrombosis [22]. Taken together, we hypothesized that cirrhotic patients with stroke might have an increased risk of portal vein thrombosis. Herein, this retrospective study was aimed at elucidating this issue.

## 2. Methods

### 2.1. Study Design

The study population was selected from our prospectively established database of cirrhotic patients without malignancy of the Department of Gastroenterology of our hospital from December 2014 to July 2019. All included patients must undergo abdominal enhanced computed tomography or magnetic resonance and endoscopy at their first enrollment. Age, sex, and the etiologies of liver cirrhosis were not limited. Repeated admissions of the same patients were excluded. Patients with abdominal surgery, including splenectomy, and splenic arterial embolization were excluded. Patients in whom a history of stroke cannot be accurately evaluated were excluded. Patients in whom the location of portal vein thrombosis cannot be evaluated due to missing images were also excluded. The study protocol was approved by the Medical Ethics Committee of our hospital. The ethical approval number was k(2019)39. The patient's informed consent was not required in our retrospective study.

### 2.2. Medical Data

The data were collected as follows.

1. Demographic information: age and sex
2. Systolic and diastolic blood pressure at admission
3. Etiologies of liver diseases: hepatitis B virus (HBV), hepatitis C virus (HCV), alcohol abuse, drug related liver diseases, Budd-Chiari syndrome, and autoimmune liver diseases, etc.
4. Clinical presentations at admission: hepatic encephalopathy, acute gastrointestinal bleeding, and ascites
5. Medical history: venous thrombus, hematological diseases, diabetes mellitus, arterial hypertension, smoking, and cardiac diseases
6. Laboratory tests: red blood cell, hemoglobin, white blood cell, platelet, total bilirubin, direct bilirubin, indirect bilirubin, albumin, alanine aminotransferase, aspartate aminotransferase, alkaline phosphatase, *γ*-glutamyl transpeptidase, blood urea nitrogen, serum, creatinine, potassium, sodium homocysteine, total cholesterol, triglyceride, high-density lipoprotein, low-density lipoprotein, lipoprotein *α*, prothrombin time, international normalized ratio (INR), activated partial thromboplastin time, D-dimer, antithrombin III, PC activity, and PS activity
7. Child-Pugh and model for the end-stage liver diseases (MELD) score

### 2.3. Definitions

Stroke was defined as previous history of stroke as well as a diagnosis of stroke at their enrollment. Type of stroke was divided into ischemic and hemorrhagic stroke according to previous medical history, clinical manifestations, and/or the results of brain imaging examination. Location of portal vein thrombosis was determined by our study group via the images of enhanced computed tomography or magnetic resonance. Portal system vessels include left portal vein (LPV), right portal vein (RPV), main portal vein (MPV), confluence of superior mesenteric vein (SMV) and splenic vein (SV), SMV, and SV.

### 2.4. Statistical Analyses

Continuous and categorical variables were expressed by median (range) and frequency (percentage), respectively. The difference between patients with and without stroke was compared using Mann-Whitney *U* test and *χ*^2^ test, as appropriate. A 1 : 1 propensity score matching (PSM) analysis was performed to adjust the effect of age, sex, Child-Pugh score, and MELD score on our statistical results. If a *p* value was less than 0.05, it would be considered statistically significant. SPSS statistical software, version 22.0 (IBM Corp, Armonk, NY, USA), was employed to perform statistical analyses.

## 3. Results

### 3.1. Patients' Characteristics

Overall, 349 cirrhotic patients were included, of whom 31 (8.88%) had stroke and 318 did not have stroke. Among the 31 patients with stroke, 29 (8.31%), 4 (1.15%), and 2 (0.57%) patients had ischemic stroke, hemorrhagic stroke, and hemorrhagic combined with ischemic stroke, respectively. The prevalence of portal vein thrombosis was 28.65% (100/349). The prevalence of thrombosis within LPV, RPV, MPV, confluence of SMV and SV, SMV, and SV was 8.31% (29/349), 9.46% (33/349), 16.05% (56/349), 11.46% (40/349), 15.19% (53/349), and 3.72% (13/349), respectively.

### 3.2. Comparison between Liver Cirrhosis with and without Stroke

Compared with the patients without stroke, those with stroke were significantly older (60.21 versus 54.21 years, *p* = 0.003) and had significantly higher proportions of alcohol abuse (64.5% versus 43.7%, *p* = 0.026), smoking (67.7% versus 42.5%, *p* = 0.007), and arterial hypertension (38.7% versus 12.9%, *p* < 0.001) and higher levels of white blood cell (4.50 versus 3.40 × 10^9^/L, *p* = 0.001) and low-density lipoprotein (2.12 versus 1.65 mmol/L, *p* = 0.013) (Table 1). There was no difference in the prevalence of portal vein thrombosis and location of portal vein thrombosis between patients with and without stroke.

### 3.3. Comparison between Cirrhotic Patients with Ischemic Stroke and Those without Stroke

Compared with the patients without stroke, those with ischemic stroke were significantly older (61.08 versus 54.21 years, *p* = 0.002) and had significantly higher proportions of alcohol abuse (65.5% versus 43.7%, *p* = 0.024), smoking (69.0% versus 42.5%, *p* = 0.006), and arterial hypertension (37.9% versus 12.9%, *p* < 0.001) and higher levels of white blood cell (4.90 versus 3.40 × 10^9^/L, *p* < 0.001), potassium (4.05 versus 3.86 mmol/L, *p* = 0.047), and low-density lipoprotein (2.12 versus 1.65 mmol/L, *p* = 0.008) (Table 2). There was no significant difference in the prevalence of portal vein thrombosis and location of portal vein thrombosis between patients with and without stroke.

### 3.4. PSM Analysis between Cirrhotic Patients with Ischemic Stroke and Those without Stroke

Twenty-eight patients were matched in each group after a 1 : 1 PSM analysis (Table 3). There was no significant difference in demographics, etiology of liver disease, laboratory tests, clinical presentations, Child-Pugh score, and MELD score between the two groups. Notably, we still did not find any significant association between ischemic stroke and portal vein thrombosis.

## 4. Discussion

Recently, a large population-based cohort study using Danish National Patient Registry demonstrated that splanchnic vein thrombosis significantly increased the risk of bleeding and arterial cardiovascular events as compared to patients with venous thromboembolism and general population [23]. In this study, splanchnic vein thrombosis referred to venous thrombosis within the portal, hepatic, mesenteric, and splenic veins; and arterial cardiovascular events included unstable angina pectoris, acute myocardial infarction, and ischemic stroke. They included 1,915 patients with splanchnic vein thrombosis, of whom 1,711 patients had portal vein thrombosis. They found that patients with splanchnic vein thrombosis had higher risk of bleeding and arterial cardiovascular events up to 1 year after diagnosis. In contrast to these findings, we did not find any significant association between stroke and portal vein thrombosis in liver cirrhosis, even in the PSM analysis. There were several potential reasons for this unexpected phenomenon. First, HCV infection, which should be regarded as a possible causative factor in the antiphospholipid syndrome with an increased prevalence of anticardiolipin antibodies [24] and an aggravated risk of hypercoagulability, is more prevalent in Western countries; by comparison, in our Chinese patients, HBV infection is the major etiology of liver cirrhosis, and the prevalence of HCV infection was only 6.02% (21/349) in our patients, which might lead to a low probability of antiphospholipid syndrome. Second, because Helicobacter pylori is an indirect risk factor of hypercoagulation and is not routinely detected in liver cirrhosis, our study did not have such data and could not compare the difference in the prevalence of Helicobacter pylori infection between patients with and without stroke. Third, our study demonstrated that the level of homocysteine was higher in patients with stroke or ischemic stroke than in patients without stroke, but the difference was not significant. This finding should be validated by expanding the number of patients who underwent laboratory tests of homocysteine levels.

Our study had several limitations. First, the number of our study population, especially patients with ischemic stroke, might not be adequate. Among the patients admitted to our Department of Gastroenterology, most of stroke events are usually asymptomatic. Therefore, in our retrospective observational study, the imaging examination of the brain has not been performed in every cirrhotic patient to evaluate the risk of asymptomatic stroke. Indeed, the imaging examination of the brain in every cirrhotic patient might not be approved by any ethical committee. Herein, the history of asymptomatic stroke, such as lacunar infarction, might be missed. Second, in the present study, we selected study population from a prospectively established database, in which only the patients undergoing enhanced computed tomography or magnetic resonance are included. Although enhanced computed tomography or magnetic resonance can evaluate more precisely the existence, extent, and degree of portal vein thrombosis, there was a potential bias in patient selection in this setting. Third, hypercoagulability is not evaluated in all patients. Fourth, Helicobacter pylori infection, which may be associated with both portal vein thrombosis and stroke, has not been studied in our study.

In conclusion, our study could not establish any association between stroke and portal vein thrombosis in liver cirrhosis. Certainly, well-designed large-scale prospective cohort studies will be necessary to confirm this finding.

## Figures and Tables

**Table 1:** Comparison between patients with and without stroke.

Variables	Stroke		No stroke		*p* value
	No. pts	Median (range) or frequency (percentage)	No. pts	Median (range) or frequency (percentage)	
Age (years)	31	60.21 (38.72-77.30)	318	54.21 (20.57-88.73)	*0.003*
Gender (male)	31	25 (80.6%)	318	230 (72.3%)	0.319
Systolic blood pressure (mmHg)	31	125.00 (90.00-173.00)	317	122.00 (83.00-193.00)	0.594
Diastolic blood pressure (mmHg)	31	75.00 (50.00-117.00)	317	75.00 (34.00-118.00)	0.844
Etiology of liver diseases					
Hepatitis B virus infection	31	10 (32.3%)	318	125 (39.3%)	0.442
Hepatitis C virus infection	31	1 (3.2%)	318	20 (6.3%)	0.494
Alcohol abuse	31	20 (64.5%)	318	139 (43.7%)	*0.026*
Drug related	31	4 (12.9%)	318	21 (6.6%)	0.194
Budd-Chiari syndrome	31	0 (0.0%)	318	1 (0.3%)	0.755
Autoimmune liver diseases	31	0 (0.0%)	318	22 (6.9%)	0.130
Clinical presentations at admission					
Hepatic encephalopathy	31	1 (3.2%)	318	8 (2.5%)	0.812
Acute gastrointestinal bleeding	31	9 (29.0%)	318	101 (31.8%)	0.755
Ascites (no/mild/moderate-severe)	31	16 (51.6%)/8 (25.8%)/7 (22.6%)	318	127 (39.9%)/108 (34.0%)/83 (26.1%)	0.440
History					
History of venous thrombus	31	0 (0.0%)	318	7 (2.2%)	0.404
History of hematological diseases	31	1 (3.2%)	318	5 (1.6%)	0.499
History of diabetes mellitus	31	8 (25.8%)	318	51 (16.0%)	0.166
History of arterial hypertension	31	12 (38.7%)	318	41 (12.9%)	*<0.001*
History of smoking	31	21 (67.7%)	318	135 (42.5%)	0.007
History of cardiac diseases	31	5 (16.1%)	318	23 (7.2%)	0.082
Laboratory tests					
Red blood cell (10^12^/L)	31	3.33 (1.15-5.20)	318	3.27 (1.45-5.46)	0.432
Hemoglobin (g/L)	31	101.00 (37.00-174.00)	318	92.50 (28.00-156.00)	0.104
White blood cell (10^9^/L)	31	4.50 (1.30-22.70)	318	3.40 (0.70-20.80)	*0.001*
Platelet (10^9^/L)	31	86.00 (37.00-377.00)	318	73.00 (19.00-470.00)	0.169
Total bilirubin (*μ*mol/L)	31	21.10 (5.70-132.70)	318	22.00 (5.20-281.10)	0.775
Direct bilirubin (*μ*mol/L)	31	8.90 (2.00-78.20)	318	9.45 (2.00-210.40)	0.920
Indirect bilirubin (*μ*mol/L)	31	11.90 (3.60-76.00)	318	11.30 (3.20-93.80)	0.677
Albumin (g/L)	31	32.50 (22.10-44.50)	316	32.35 (14.20-50.60)	0.482
Alanine aminotransferase (U/L)	31	20.06 (7.74-176.68)	318	24.34 (4.23-613.24)	0.396
Aspartate aminotransferase (U/L)	31	30.73 (9.74-143.00)	318	34.33 (9.63-761.63)	0.824
Alkaline phosphatase (U/L)	31	88.23 (28.83-337.00)	318	94.40 (31.00-983.93)	0.641
*γ*-Glutamyl transpeptidase (U/L)	31	70.00 (10.00-1779.18)	318	42.64 (7.54-1283.02)	0.121
Blood urea nitrogen (mmol/L)	31	5.85 (3.52-47.25)	314	5.31 (0.64-24.80)	0.226
Serum creatinine (*μ*mol/L)	31	70.90 (42.82-267.63)	314	64.07 (23.83-178.55)	0.228
Potassium (mmol/L)	31	4.01 (2.80-5.41)	317	3.86 (2.42-5.28)	0.066
Sodium (mmol/L)	31	138.00 (134.10-145.50)	317	138.90 (118.00-152.90)	0.574
Homocysteine (*μ*mol/L)	16	10.49 (6.70-31.79)	170	9.16 (1.59-102.81)	0.145
Total cholesterol (mmol/L)	20	3.68 (1.82-6.58)	208	3.11 (1.14-6.29)	0.148
Triglyceride (mmol/L)	20	0.83 (0.41-6.22)	208	0.85 (0.35-4.81)	0.661
High-density lipoprotein (mmol/L)	20	0.93 (0.47-1.39)	208	0.94 (0.24-2.29)	0.766
Low-density lipoprotein (mmol/L)	20	2.12 (0.99-4.37)	208	1.65 (0.47-4.06)	*0.013*
Lipoprotein *α* (mg/L)	20	88.70 (17.90-466.40)	208	63.20 (3.60-911.40)	0.108
Prothrombin time (seconds)	31	15.30 (12.70-20.40)	314	15.70 (10.30-28.00)	0.188
International normalized ratio	31	1.23 (0.99-1.75)	314	1.27 (0.89-2.77)	0.164
Activated partial thromboplastin time (seconds)	31	40.20 (19.80-53.80)	314	39.90 (23.10-71.30)	0.808
D-dimer (mg/L)	19	1.32 (0.16-10.56)	252	0.86 (0.10-46.17)	0.873
Antithrombin III (%)	11	65.30 (40.00-84.00)	118	63.00 (21.00-123.00)	0.933
Protein C activity (%)	6	57.30 (49.80-75.10)	54	59.65 (24.00-119.30)	0.721
Protein S activity (%)	6	61.55 (55.60-73.30)	54	63.50 (20.70-123.60)	0.873
Child-Pugh score	31	7.00 (5.00-10.00)	313	7.00 (5.00-13.00)	0.837
MELD score	31	10.46 (7.23-18.00)	312	10.41 (6.43-30.03)	0.736
Portal vein thrombosis	31	8 (25.8%)	318	92 (28.9%)	0.713
LPV		4 (12.9%)		25 (7.9%)	0.332
RPV		2 (6.5%)		31 (9.7%)	0.549
MPV		4 (12.9%)		52 (16.4%)	0.617
Confluence of SMV and SV		5 (16.1%)		35 (11.0%)	0.393
SMV		2 (6.5%)		51 (16.0%)	0.156
SV		0 (0.0%)		13 (4.1%)	0.251

No. pts: number of patients; MELD: model for the end-stage liver diseases; LPV: left portal vein; RPV: right portal vein; MPV: main portal vein; SMV: superior mesenteric vein; SV: splenic vein.

**Table 2:** Comparison between patients with ischemic stroke and those without stroke.

Variables	Ischemic stroke		No stroke		*p* value
	No. pts	Median (range) or frequency (percentage)	No. pts	Median (range) or frequency (percentage)	
Age (years)	29	61.08 (38.72-77.30)	318	54.21 (20.57-88.73)	*0.002*
Gender (male)	29	23 (79.3%)	318	230 (72.3%)	0.418
Systolic blood pressure (mmHg)	29	124.00 (90.00-173.00)	317	122.00 (83.00-193.00)	0.975
Diastolic blood pressure (mmHg)	29	73.00 (50.00-108.00)	317	75.00 (34.00-118.00)	0.700
Etiology of liver diseases					
Hepatitis B virus infection	29	10 (34.5%)	318	125 (39.3%)	0.610
Hepatitis C virus infection	29	1 (3.4%)	318	20 (6.3%)	0.539
Alcohol abuse	29	19 (65.5%)	318	139 (43.7%)	*0.024*
Drug related	29	4 (13.8%)	318	21 (6.6%)	0.152
Budd-Chiari syndrome	29	0 (0.0%)	318	1 (0.3%)	0.762
Autoimmune liver diseases	29	0 (0.0%)	318	22 (6.9%)	0.143
Clinical presentations at admission					
Hepatic encephalopathy	29	1 (3.4%)	318	8 (2.5%)	0.762
Acute gastrointestinal bleeding	29	8 (27.6%)	318	101 (31.8%)	0.643
Ascites (no/mild/moderate-severe)	29	15 (51.7%)/7 (24.1%)/7 (24.1%)	318	127 (39.9%)/108 (34.0%)/83 (26.1%)	0.424
History					
History of venous thrombus	29	0 (0.0%)	318	7 (2.2%)	0.420
History of hematological diseases	29	1 (3.4%)	318	5 (1.6%)	0.458
History of diabetes mellitus	29	8 (27.6%)	318	51 (16.0%)	0.113
History of arterial hypertension	29	11 (37.9%)	318	41 (12.9%)	*<0.001*
History of smoking	29	20 (69.0%)	318	135 (42.5%)	*0.006*
History of cardiac diseases	29	5 (17.2%)	318	23 (7.2%)	0.058
Laboratory tests					
Red blood cell (10^12^/L)	29	3.33 (1.15-5.20)	318	3.27 (1.45-5.46)	0.471
Hemoglobin (g/L)	29	101.00 (37.00-174.00)	318	92.50 (28.00-156.00)	0.126
White blood cell (10^9^/L)	29	4.90 (1.30-22.70)	318	3.40 (0.70-20.80)	*<0.001*
Platelet (10^9^/L)	29	90.00 (37.00-377.00)	318	73.00 (19.00-470.00)	0.068
Total bilirubin (*μ*mol/L)	29	25.10 (5.70-132.70)	318	22.00 (5.20-281.10)	0.606
Direct bilirubin (*μ*mol/L)	29	9.00 (2.00-78.20)	318	9.45 (2.00-210.40)	0.892
Indirect bilirubin (*μ*mol/L)	29	12.40 (3.60-76.00)	318	11.30 (3.20-93.80)	0.576
Albumin (g/L)	29	31.10 (22.10-44.50)	316	32.35 (14.20-50.60)	0.401
Alanine aminotransferase (U/L)	29	21.33 (7.74-176.68)	318	24.34 (4.23-613.24)	0.643
Aspartate aminotransferase (U/L)	29	31.44 (9.74-143.00)	318	34.33 (9.63-761.63)	0.902
Alkaline phosphatase (U/L)	29	92.49 (28.83-337.00)	318	94.40 (31.00-983.93)	0.921
*γ*-Glutamyl transpeptidase (U/L)	29	70.00 (10.00-1779.18)	318	42.64 (7.54-1283.02)	0.073
Blood urea nitrogen (mmol/L)	29	5.82 (3.52-47.25)	314	5.31 (0.64-24.80)	0.271
Serum creatinine (*μ*mol/L)	29	70.90 (42.82-267.63)	314	64.07 (23.83-178.55)	0.253
Potassium (mmol/L)	29	4.05 (2.80-5.41)	317	3.86 (2.42-5.28)	*0.047*
Sodium (mmol/L)	29	137.90 (134.10-145.50)	317	138.90 (118.00-152.90)	0.430
Homocysteine (*μ*mol/L)	15	10.47 (6.70-31.79)	170	9.16 (1.59-102.81)	0.179
Total cholesterol (mmol/L)	19	3.77 (1.82-6.58)	208	3.11 (1.14-6.29)	0.104
Triglyceride (mmol/L)	19	0.86 (0.47-6.22)	208	0.85 (0.35-4.81)	0.414
High-density lipoprotein (mmol/L)	19	0.92 (0.47-1.39)	208	0.94 (0.24-2.29)	0.762
Low-density lipoprotein (mmol/L)	19	2.12 (0.99-4.37)	208	1.65 (0.47-4.06)	*0.008*
Lipoprotein *α* (mg/L)	19	91.70 (17.90-466.40)	208	63.20 (3.60-911.40)	0.114
Prothrombin time (seconds)	29	15.30 (12.70-20.40)	314	15.70 (10.30-28.00)	0.198
International normalized ratio	29	1.23 (0.99-1.75)	314	1.27 (0.89-2.77)	0.170
Activated partial thromboplastin time (seconds)	29	40.00 (19.80-53.80)	314	39.90 (23.10-71.30)	0.952
D-dimer (mg/L)	19	1.32 (0.16-10.56)	252	0.86 (0.10-46.17)	0.873
Antithrombin III (%)	11	65.30 (40.00-84.00)	118	63.00 (21.00-123.00)	0.933
Protein C activity (%)	6	57.30 (49.80-75.10)	54	59.65 (24.00-119.30)	0.721
Protein S activity (%)	6	61.55 (55.60-73.30)	54	63.50 (20.70-123.60)	0.873
Child-Pugh score	29	7.00 (5.00-10.00)	313	7.00 (5.00-13.00)	0.749
MELD score	29	10.46 (7.23-18.00)	312	10.41 (6.43-30.03)	0.892
Portal vein thrombosis	29	7 (24.1%)	318	92 (28.9%)	0.584
LPV		3 (10.3%)		25 (7.9%)	0.638
RPV		2 (6.9%)		31 (9.7%)	0.616
MPV		4 (13.8%)		52 (16.4%)	0.720
Confluence of SMV and SV		5 (17.2%)		35 (11.0%)	0.314
SMV		2 (6.9%)		51 (16.0%)	0.190
SV		0 (0.0%)		13 (4.1%)	0.267

No. pts: number of patients; MELD: model for the end-stage liver diseases; LPV: left portal vein; RPV: right portal vein; MPV: main portal vein; SMV: superior mesenteric vein; SV: splenic vein.

**Table 3:** Characteristics of cirrhotic patients with ischemic stroke and those without stroke after propensity score matching.

Variables	Overall (*n* = 56)	Ischemic stroke (*n* = 28)	No stroke (*n* = 28)	*p* value
Age (years)	59.68 (35.18-77.30)	60.65 (38.72-77.30)	58.44 (35.18-75.72)	0.258
Gender (male)	47 (83.9%)	22 (78.6%)	25 (89.3%)	0.275
Systolic blood pressure (mmHg)	125.00 (83.00-173.00)	124.50 (90.00-173.00)	126.50 (83.00-158.00)	0.491
Diastolic blood pressure (mmHg)	75.50 (44.00-108.00)	72.00 (50.00-108.00)	81.00 (44.00-107.00)	0.161
Etiology of liver diseases				
Hepatitis B virus infection	23 (41.1%)	10 (35.7%)	13 (46.4%)	0.415
Hepatitis C virus infection	1 (1.8%)	1 (3.6%)	0 (0.0%)	0.313
Alcohol abuse	35 (62.5%)	18 (64.3%)	17 (60.7%)	0.783
Drug related	6 (10.7%)	4 (14.3%)	2 (7.1%)	0.388
Budd-Chiari syndrome	0 (0.0%)	0 (0.0%)	0 (0.0%)	NA
Autoimmune liver diseases	1 (1.8%)	0 (0.0%)	1 (3.6%)	0.313
Clinical presentations at admission				
Hepatic encephalopathy	2 (3.6%)	1 (3.6%)	1 (3.6%)	1.000
Acute gastrointestinal bleeding	13 (23.2%)	7 (25.0%)	6 (21.4%)	0.752
Ascites (no/mild/moderate-severe)	27 (48.2%)/14 (25.0%)/15 (26.8%)	15 (53.6%)/6 (21.4%)/7 (25.0%)	12 (42.9%)/8 (28.6%)/8 (28.6%)	0.710
History				
History of venous thrombus	0 (0.0%)	0 (0.0%)	0 (0.0%)	NA
History of hematological diseases	1 (1.8%)	1 (3.6%)	0 (0.0%)	0.313
History of diabetes mellitus	12 (21.4%)	8 (28.6%)	4 (14.3%)	0.193
History of arterial hypertension	13 (23.2%)	10 (35.7%)	3 (10.7%)	0.027
History of smoking	39 (69.6%)	20 (71.4%)	19 (67.9%)	0.771
History of cardiac diseases	8 (14.3%)	5 (17.9%)	3 (10.7%)	0.445
Laboratory tests				
Red blood cell (10^12^/L)	3.52 (1.15-5.20)	3.34 (1.15-5.20)	3.69 (1.74-5.08)	0.456
Hemoglobin (g/L)	109.00 (37.00-174.00)	102.00 (37.00-174.00)	110.50 (58.00-156.00)	0.812
White blood cell (10^9^/L)	4.25 (1.30-22.70)	4.70 (1.30-22.70)	4.05 (1.30-20.80)	0.063
Platelet (10^9^/L)	84.50 (37.00-423.00)	88.00 (37.00-377.00)	75.00 (37.00-423.00)	0.961
Total bilirubin (*μ*mol/L)	24.25 (5.70-132.70)	25.30 (5.70-132.70)	24.15 (6.60-47.00)	0.718
Direct bilirubin (*μ*mol/L)	9.55 (2.00-78.20)	9.30 (2.00-78.20)	9.60 (2.50-27.00)	0.961
Indirect bilirubin (*μ*mol/L)	12.45 (3.60-76.00)	12.60 (3.60-76.00)	12.35 (4.10-27.70)	0.967
Albumin (g/L)	32.55 (22.10-46.20)	30.75 (22.10-44.50)	33.95 (24.50-46.20)	0.201
Alanine aminotransferase (U/L)	24.79 (7.53-176.68)	21.66 (7.74-176.68)	25.59 (7.53-140.00)	0.793
Aspartate aminotransferase (U/L)	33.94 (9.74-166.49)	34.22 (9.74-143.00)	33.94 (13.94-166.49)	0.857
Alkaline phosphatase (U/L)	98.86 (28.83-337.00)	93.60 (28.83-337.00)	102.83 (52.28-337.00)	0.481
*γ*-Glutamyl transpeptidase (U/L)	60.87 (10.00-1779.18)	69.50 (10.00-1779.18)	56.00 (10.93-552.26)	0.611
Blood urea nitrogen (mmol/L)	5.62 (2.96-47.25)	5.81 (3.52-47.25)	5.38 (2.96-20.15)	0.502
Serum creatinine (*μ*mol/L)	66.64 (42.82-267.63)	68.96 (42.82-267.63)	64.19 (44.50-117.53)	0.870
Potassium (mmol/L)	3.99 (2.74-5.41)	4.03 (2.80-5.41)	3.89 (2.74-4.75)	0.517
Sodium (mmol/L)	138.50 (133.50-145.50)	137.95 (134.10-145.50)	139.65 (133.50-145.20)	0.210
Prothrombin time (seconds)	15.30 (12.70-20.40)	15.30 (12.70-20.40)	15.25 (12.00-18.90)	0.774
International normalized ratio	1.24 (0.93-1.75)	1.24 (0.99-1.75)	1.24 (0.93-1.64)	0.883
Activated partial thromboplastin time (seconds)	40.10 (19.80-53.80)	40.10 (19.80-53.80)	40.30 (27.50-50.80)	0.961
Child-Pugh score	7.00 (5.00-10.00)	7.00 (5.00-10.00)	7.00 (5.00-13.00)	0.259
MELD score	10.43 (6.43-18.00)	10.52 (7.23-18.00)	10.32 (6.43-15.58)	0.676
Portal vein thrombosis	14 (25.0%)	7 (25.0%)	7 (25.0%)	1.000

MELD: model for the end-stage liver diseases.

## Data Availability

The datasets generated during and/or analyzed during the current study are available from the corresponding author on reasonable request.

## Abbreviations

FVIII: Factor VIII
PC: Protein C
MTHFR: Methylenetetrahydrofolate reductase
HBV: Hepatitis B virus
HCV: Hepatitis C virus
INR: International normalized ratio
MELD: Model for the end-stage liver diseases
LPV: Left portal vein
RPV: Right portal vein
MPV: Main portal vein
SMV: Superior mesenteric vein
SV: Splenic vein
PSM: Propensity score matching.
